# A Retrospective Study Assessing the Outcomes of Immediate Prepectoral and Subpectoral Implant and Mesh-Based Breast Reconstruction

**DOI:** 10.3390/cancers14133188

**Published:** 2022-06-29

**Authors:** Thomas Wow, Agnieszka Kolacinska-Wow, Mateusz Wichtowski, Katarzyna Boguszewska-Byczkiewicz, Zuzanna Nowicka, Katarzyna Ploszka, Karolina Pieszko, Dawid Murawa

**Affiliations:** 1 Department of General Surgery and Surgical Oncology, Faculty of Medicine and Health Sciences, University of Zielona Gora, Zyty 26, 65-046 Zielona Gora, Poland; drthomaswow@gmail.com (T.W.); mawichto@gmail.com (M.W.); saurie@gmail.com (K.P.); dmurawa@gmail.com (D.M.); 2 Department of Oncological Physiotherapy, Medical University of Lodz, Paderewskiego 4, 93-509 Lodz, Poland; 3 Breast Cancer Unit, Department of Surgical Oncology, Cancer Center, Copernicus Memorial Hospital, Paderewskiego 4, 93-509 Lodz, Poland; kkatarzynabboguszewska@gmail.com; 4 Department of Biostatistics and Translational Medicine, Medical University of Lodz, Kosciuszki 4, 92-215 Lodz, Poland; zuzanna.nowicka@umed.lodz.pl (Z.N.); katarzyna.ploszka@stud.umed.lodz.pl (K.P.); 5 Department of Plastic Surgery and Burns, Hospital of Nowa Sol, Chalubinskiego 7, 67-100 Nowa Sol, Poland

**Keywords:** breast reconstruction, synthetic mesh

## Abstract

**Simple Summary:**

The aim of the study is to compare the outcomes of implant and synthetic TIGR mesh-based breast reconstructions in breast cancer patients and women, who have higher lifetime risk for breast cancer, with mutated (changed) copy of genes: BRCA1, BRCA2, PALB2 or CHEK2. This study included 170 patients after 232 mastectomies and immediate breast reconstructions. Preoperative chemotherapy was associated with more frequent minor complications, but not major ones, while postoperative chemotherapy was related to more frequent serious postoperative complications. Postoperative radiotherapy was associated with a higher rate of minor complications than no-radiotherapy. Our research found complications to be significantly associated with expander, big- volume breast, mastectomy in breast cancer patients vs risk-reducing mastectomy/prophylactic surgery in women with mutated gene and postoperative chemotherapy. Patients in whom prepectoral (prosthesis is placed above the pectoralis major muscle) surgeries were performed demonstrated shorter hospitalization time, and lower minor complication rates, but similar major complication rates. Implant-based breast reconstruction with the use of synthetic mesh is a safe and effective method of breast restoration, associated with low morbidity and good cosmesis. Nevertheless, further studies are needed to evaluate the benefits of such treatments.

**Abstract:**

(1) Introduction: In response to patient concerns about breast cancer recurrence, increased use of breast magnetic resonance imaging and genetic testing, and advancements in breast reconstruction techniques, mastectomy rates have been observed to rise over the last decade. The aim of the study is to compare the outcomes of prepectoral and subpectoral implants and long-term, dual-stage resorbable mesh-based breast reconstructions in mutation carriers (prophylactic surgery) and breast cancer patients. (2) Patients and methods: This retrospective, two-center study included 170 consecutive patients after 232 procedures: Prepectoral surgery was performed in 156 cases and subpectoral was performed in 76. (3) Results: Preoperative chemotherapy was associated with more frequent minor late complications (*p* < 0.001), but not major ones (*p* = 0.101), while postoperative chemotherapy was related to more frequent serious (*p* = 0.005) postoperative complications. Postoperative radiotherapy was associated with a higher rate of minor complications (31.03%) than no-radiotherapy (12.21%; *p* < 0.001). Multivariate logistic regression found complications to be significantly associated with an expander (OR = 4.43), skin-reducing mastectomy (OR = 9.97), therapeutic mastectomy vs. risk-reducing mastectomy (OR = 4.08), and postoperative chemotherapy (OR = 12.89). Patients in whom prepectoral surgeries were performed demonstrated significantly shorter median hospitalization time (*p* < 0.001) and lower minor complication rates (5.77% vs. 26.32% *p* < 0.001), but similar major late complication rates (*p* = 0.915). (4) Conclusions: Implant-based breast reconstruction with the use of long-term, dual-stage resorbable, synthetic mesh is a safe and effective method of breast restoration, associated with low morbidity and good cosmesis. Nevertheless, prospective, multicenter, and long-term outcome data studies are needed to further evaluate the benefits of such treatments.

## 1. Introduction

The most common type of malignancy diagnosed in women worldwide is breast cancer, with a global annual mortality rate of 685,000, and an incidence of 2.3 million in 2020 [1]. In patients with early breast cancer, the gold standard in treatment is breast-conserving surgery [2,3]. However, due to patient concerns about breast recurrence, increased use of breast magnetic resonance imaging (MRI) and genetic testing, and advancements in breast reconstruction techniques, mastectomy rates have been observed to rise over the last decade [4], somewhat paradoxically in an era of de-escalation therapy. Among these reconstruction techniques, we can distinguish the following:

- Autologous (pedicled or free flaps, fat transfer) and allogeneic/alloplastic (implant-based, synthetic meshes, acellular dermal matrix ADM) breast reconstructions.
- Immediate (at the same time as mastectomy) and delayed (performed months or years after mastectomy) breast reconstructions.
- One-stage (direct-to-implant DTI) or two-stage (expander first and then permanent implant) breast reconstructions.
- Prepectoral reconstructions, a pectoralis-major-sparing technique where the prosthesis is placed above the muscle, usually with mesh or ADM coverage, and subpectoral reconstructions, where the breast implant is placed partially behind the pectoralis major muscle and partially behind the lower mastectomy skin flap, with or without lower pole reinforcement using mesh or ADM [5,6,7].

Prepectoral breast reconstruction avoids animation deformity, reduces postoperative pain and convalescence, and may be more tolerant to post-mastectomy radiotherapy. A prepectoral implant is also unaffected by pectoralis muscle fibrosis: When the pectoralis major muscle is radiated, it becomes fibrotic and shortens, thus elevating any underlying implant. However, one unwanted sequela of the procedure is the tightening of the skin envelope. It has been proposed that complete prosthesis coverage with ADM, sparing the pectoralis major, may provide greater protection against the adverse effect of radiotherapy compared to partial coverage [8]. Prepectoral breast reconstruction also achieves favorable aesthetic outcomes and seems to limit capsular contracture. However, it significantly increases the cost of surgery due to inter alia the use of meshes and ADMs [7].

Prepectoral breast reconstruction is not a new concept and was routinely carried out in the 1970s and 1980s. However, its use was linked to a high rate of complications, such as explantation and capsular contracture. Over the last decade, significant advancements have enabled breast surgeons to reconceptualize the concept of prepectoral breast reconstructions, and modern procedures include better surgical techniques such as nipple-sparing mastectomy (NSM), modern implants, meshes and ADMs, autologous fat grafting, and sophisticated tissue perfusion technologies [9,10,11].

The aim of the study is to compare the outcomes of prepectoral and subpectoral implant and long-term, dual-stage resorbable mesh-based breast reconstructions in mutation carriers (prophylactic surgery) and breast cancer patients.

## 2. Patients and Methods

This retrospective, two-center study included 170 consecutive patients undergoing 232 procedures. All were operated on in the Department of Surgical Oncology, Breast Cancer Unit in Copernicus Memorial Hospital, Cancer Center, Medical University of Lodz, Poland and the Department of Surgical Oncology in Zielona Gora, Poland between March 2019 and October 2021. The follow-up proceeded until 30 November 2021. Inclusion criteria comprised the following: Age over 18, diagnosis of breast cancer or the presence of BRCA1, BRCA2, PALB2, or CHEK2 mutation, the use of nipple-sparing (NSM) or skin-sparing (SSM) or skin-reducing mastectomy (SRM) with immediate breast reconstruction (IBR): Implant-based with synthetic long-term, dual-stage resorbable TIGR™ mesh (in all cases) placed prepectoral or subpectoral, direct-to-implant one-stage or expander-to-implant two-stage. The exclusion criteria comprised the following: Patients with other cancers, male breast cancer patients, pregnant women, patients with delayed breast reconstructions, and patients with autologous breast reconstructions.

Concerning patient-reported outcome measures (PROMs), patients used the rating scale from 1 to 5 and completed the form 4 weeks after surgery.

Ethics committee approval was obtained from the Institutional Review Board, Collegium Medicum University of Zielona Gora, number RCM-CM-KBUZ. The statement from the President of the Ethics Committee, Collegium Medicum of Zielona Gora, Prof. J. Hiszkiewicz: “We declare that the study described in the application is not a medical experiment and does not require the opinion of the Bioethical Commission”. Number RCM-CM-KBUZ. Date: 16 February 2022.

## 3. Statistical Analysis

For nominal variables, 2 × 2 tables with counts and percentages were used to assess the differences between study groups, and the statistical significance was determined using the Chi2 test with appropriate corrections. For continuous variables, the normality of distribution was confirmed with the Shapiro–Wilk test, and based on the result, the Mann–Whitney U-test was used to assess the differences between the groups. This test was also used for ordinal variables. Both continuous and ordinal variables were described using median with 25 and 75% quartile.

As several patients included in the study had undergone operations for both breasts, i.e., not all observations were independent, the assumptions of some statistical tests were not met; however, as the maximum number of non-independent observations per patient was two, and 108 patients had only received one procedure, compared to 62 with both breasts operated, the tests were nevertheless used in the study. A sensitivity analysis was performed to evaluate the potential effect of this violation by randomly excluding one additional observation per patient.

A composite endpoint was adopted, defined as the occurrence of minor or major complications. Univariate logistic regression was used to determine the statistical significance of risk factors influencing the composite endpoint. Multivariate logistic regression and logistic regression with stepwise backward feature elimination were used to assess the simultaneous effect of multiple variables on the endpoint. An odds ratio (OR) with a 95% confidence interval (95% CI) was used as an effective measure for the univariate and multivariate logistic regression. For the regression model with backward feature elimination, a *p*-value of 0.15 was used as a cut-off. The model quality was assessed by the area under the ROC curve, with a 95% confidence interval, as well as the sensitivity and specificity of the test. The optimal cut-off value was determined using the Youden index. Statistical significance was assessed at *p* < 0.05. STATISTICA software version 13.1 (TIBCO Software 2022, Palo Alto, CA, USA) was used for statistical analysis.

## 4. Results

In total, 170 patients were included in the study. Two hundred and thirty-two procedures were performed, with prepectoral surgery in 156 cases (67.24%) and subpectoral in 76 (32.76%). The median follow-up for all patients was 20 months (range: 1–33 months). Patient and procedure-related characteristics are presented in Table 1 and Table 2. The weight of the specimen ranged from 150 g to 840 g, and the volume of the implant from 195 mL to 685 mL. SRMs were performed in 17 patients (Table 2). Three patients (1.7%) demonstrated complications during hospitalization (e.g., hematoma, which required surgical revision). The length of the hospital stay was three days in five cases (2.94%), four days in 126 cases (74.12%), five days in 18 cases (10.59%), six days in 10 (5.88%), seven days in 9 (5.29%), and eight days in 2 cases (1.18%).

In addition, minor and major complications were observed after discharge from the hospital. Minor complications occurred after 29 (12.50%) surgeries, including 5.77% of prepectoral and 26.32% of subpectoral procedures (*p* < 0.001). These complications included superficial necrosis in 13 cases (5.60%), infection in 10 (4.31%), and erythema in 1 case (0.43%).

**Figure 1:**
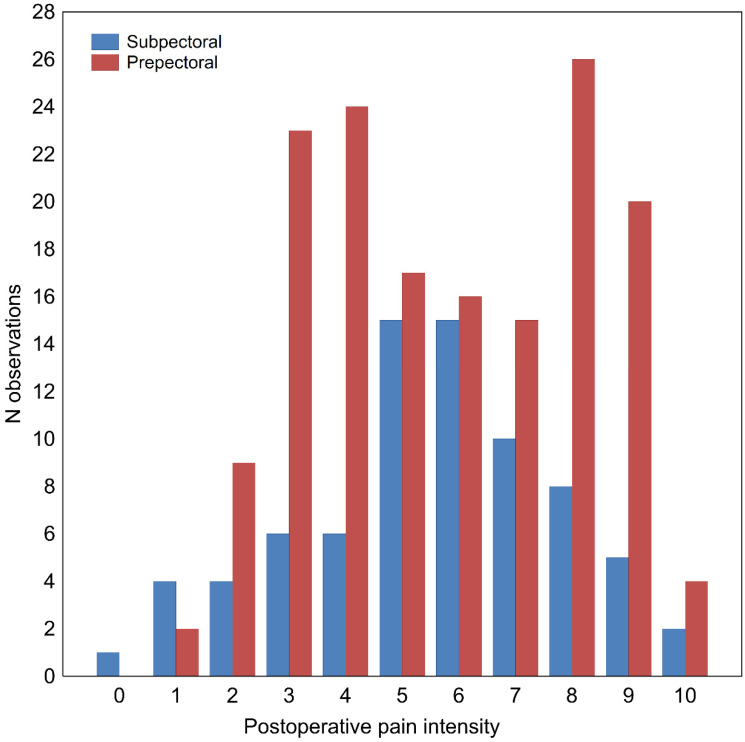
Distribution of postoperative pain intensity on 11-point numeric rating scale in patients undergoing subpectoral or prepectoral breast reconstruction surgery. Postoperative pain intensity, categorized by type of surgery performed (prepectoral vs. subpectoral).

Major complications occurred in 24 (10.34%) patients. These complications included full-thickness skin necrosis 8 (3.45%) and implant loss in 19 patients (8.19%), including 17 (10.90%) prepectoral vs. 7 (9.21%) subpectoral, as well as severe infection in 4 patients (1.72%) and skin fistula in 1 patient (0.43%). Postoperative pain intensity was recorded on the 11-point NRS (numeric rating scale), ranging from 0 (no pain) to 10 (worst pain imaginable), and the results are presented in Figure 1. None of the patients suffered from diabetes.

Prepectoral (*n* = 156) and subpectoral (*n* = 76) breast reconstruction surgeries, as well as therapeutic (TM, *n* = 109) and risk-reducing mastectomies (RRM, *n* = 123), were compared with regard to therapeutic outcomes. To account for the non-independence of some observations, such as in the case of patients who had undergone operations for both breasts, a sensitivity analysis was performed where only one observation was randomly chosen per patient; this yielded very similar results (not shown).

**Figure 2:**
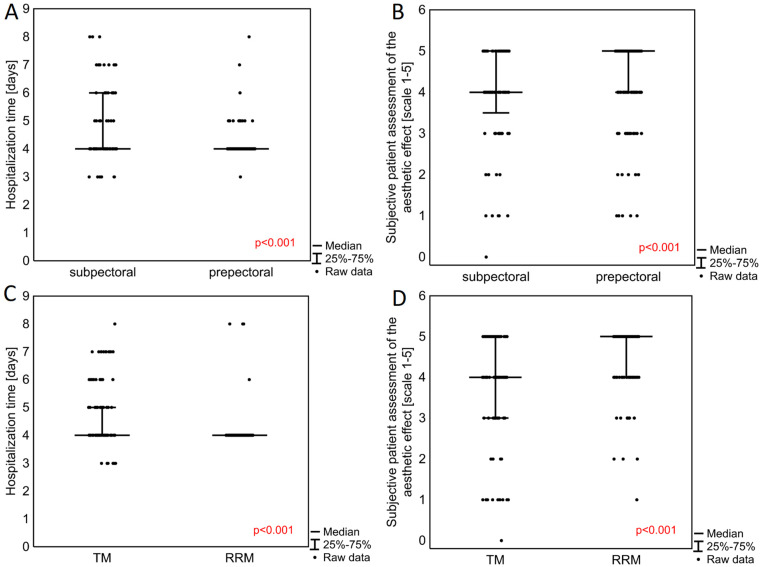
Hospitalization time (**A**) and subjective patient assessment of the aesthetic effect of surgery (**B**) in patients undergoing subpectoral vs. prepectoral breast reconstruction technique and in patients undergoing therapeutic (TM) or risk-reducing mastectomy (RRM) (**C**,**D**). *p*-values obtained with Mann–Whitney U test. Box plots comparing the duration of hospitalization after the procedure, in days, and the subjective patient assessment of the final effect (patient-reported aesthetic outcome) on a scale from 1 to 5, according to subpectoral vs. prepectoral breast reconstructions (**A**,**C**), and also type of surgery, i.e., TM vs. RRM (**B**,**D**).

Patients operated on with the prepectoral method also demonstrated a significantly shorter median hospitalization time (*p* < 0.001) (Table 2, Figure 2A). Hospitalization time was also shorter in patients operated on with RRM in contrast to TM (*p* < 0.001) (Figure 2B). Patients undergoing prepectoral surgery also reported significantly higher post-operative patient-reported aesthetic outcomes on a scale of 1 to 5 (*p* < 0.001) (5.00, 25–75%: 4.00–5.00) compared to those receiving subpectoral breast reconstructions (4.00, 25–75%: 3.50–5.00) (Figure 2C). RRM was associated with a significantly better final aesthetic effect (*p* < 0.001), with a median score of 5.00 (4.00–5.00) compared with 4.00 (3.00–5.00) for TM (Figure 2D).

Minor complication rates were noted more often in the subpectoral (26.32%) than the prepectoral (5.77%) group (*p* < 0.001) (Table 2). However, the rate of major late complications did not differ significantly (*p* = 0.869) between the groups. Preoperative chemotherapy was associated with a significantly more frequent occurrence of minor postoperative complications (*p* < 0.001), but not major ones (*p* = 0.101, Supplementary Materials Tables S1 and S2). Postoperative chemotherapy was associated with a more frequent occurrence of only serious postoperative complications (*p* = 0.005) (Supplementary Materials Tables S1 and S2).

Postoperative radiotherapy was also associated with a higher rate of minor complications: 31.03% in radiotherapy vs. 7.39% in no-radiotherapy (*p* < 0.001, Supplementary Materials Table S2). No such difference was noted for preoperative radiotherapy (*p* > 0.999). No statistically significant difference in the occurrence of late major complications was found between the radiotherapy and no-radiotherapy groups (Supplementary Materials Tables S1 and S2).

Longer hospitalization time was also noted in breast cancer patients who received therapeutic mastectomies compared to mutation carriers (prophylactic surgery) in whom risk-reducing mastectomies were carried out (*p* < 0.001). No difference in postoperative pain was found between the therapeutic and risk-reducing mastectomy (prophylactic mastectomy) groups (*p* = 0.450, Table 3).

## 5. Univariate and Multivariate Logistic Regression

The influence of the following statistically significant factors on the development of the composite endpoint, defined as the occurrence of minor complications or major complications, including the presence of seroma, was determined using univariate logistic regression (Table 4). These factors comprised age (OR = 1.06, 95% CI 1.02–1.09), BMI (OR = 1.22, 95% CI 1.11–1.33), smoking (OR = 17.63, 95% CI: 2.12–140.22), expander or permanent implant placement (OR = 4.95, 95% CI: 2.62–9.37), SSM performed (OR = 2.34, 95% CI 1.22–4.48), SRM performed (OR = 5.83, 95% CI: 1.84–18.49), preoperative chemotherapy (OR = 2.98, 95% CI: 1.66–5.34), postoperative chemotherapy (OR = 16.30, 95% CI: 3.67–72.50), postoperative radiotherapy (OR = 2.43, 95% CI: 1.03–5.74), and TM vs. RRM (OR = 4.87, 95% CI: 2.75–8.63). For TM patients, Stage I was a factor that influenced the development of the composite endpoint (OR = 0.12, 95% CI: 0.02–0.71).

After introducing the same factors into the multivariate model (Table 5), only smoking (OR = 10.53, 95% CI: 1.07–103.86), reconstruction method—expander (OR = 4.70, 95% CI: 1.39–15.91), SRM mastectomy performed (OR = 12.86, 95% CI: 2.93–56.51), and postoperative chemotherapy (OR = 16.72, 95% CI: 3.27–85.53) were found to significantly increase the chance of developing the composite endpoint, but not the prepectoral vs. subpectoral technique (*p* = 0.578). Separate analyses were performed in TM, also accounting for tumor histology and stage (Supplementary Materials Table S3), and in RRM (Supplementary Materials Table S4).

**Figure 3:**
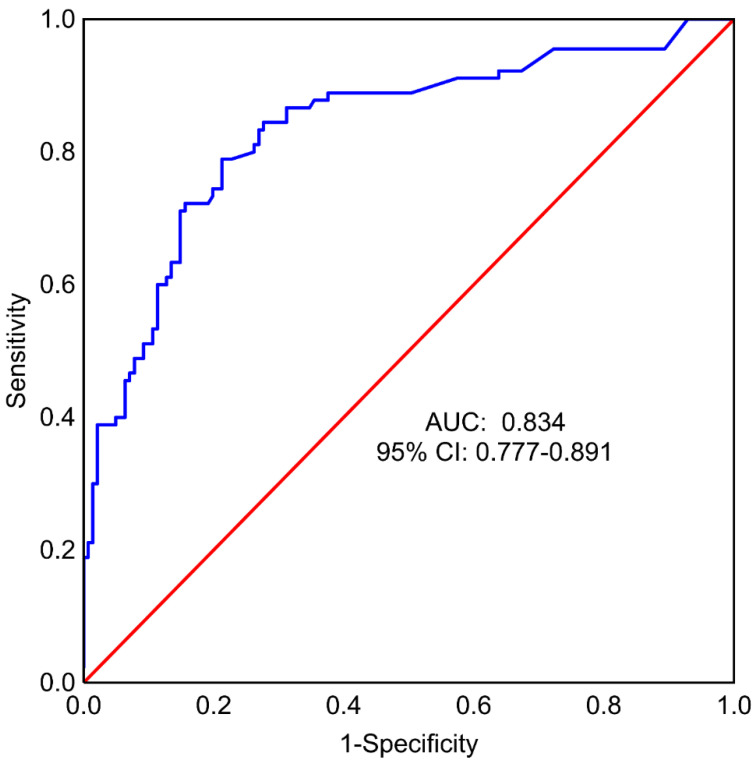
Receiver operating characteristic (ROC) curve for multivariate model built using backward feature selection for predicting minor and major complications following breast reconstruction surgery. AUC—area under the ROC curve, 95% CI −95% confidence interval. Confidence intervals were calculated using formula by Hanley and McNeil. Model quality assessment using the ROC curve.

A multivariate regression model with backward stepwise feature selection was also prepared to identify a minimal set of variables useful for predicting surgery complications, using all factors considered for the whole model as input: Age, BMI, specimen weight, implant size, smoking, method of reconstruction, SSM vs. NSM, SRM, pre- and post-operative chemotherapy and radiotherapy, and types of surgery: TM vs. RRM. In this model, the following factors were found to significantly affect the occurrence of the endpoint: The use of expander vs. implant (OR = 4.43, 95% CI: 1.40–14.01), postoperative chemotherapy (OR = 12.89, 95% CI: 2.60–63.98), type of mastectomy—SRM (OR = 9.97, 95% CI: 2.52–39.35), and types of surgery: TM vs. RRM (OR = 4.08, 95% CI: 1.85–9.04) (Table 6). Other features included in the model were BMI, preoperative radiotherapy, type of mastectomy (SSM vs. NSM), and smoking. The area under the ROC curve (AUC) for the model was equal to 0.834 (95% CI: 0.777–0.891) (Figure 3). Sensitivity was 78.90% and specificity was 78.70% (cut-off = 0.39).

## 6. Discussion

This retrospective, two-center study compared outcomes of immediate alloplastic breast reconstruction with the long-term, dual-stage resorbable synthetic mesh TIGR Matrix^®^ (Novus Scientific, Uppsala, Sweden). The TIGR is less expensive than biological meshes, but with a similar rate of postsurgical complication [12,13,14,15]. We analyzed subgroups of:

- Therapeutic (in breast cancer patients) and risk-reducing (in mutation carriers) mastectomies.
- Prepectoral and subpectoral breast reconstructions.
- Immediate one-stage direct-to-implant and immediate two-stage expander-to-implant breast reconstructions.

The area under the ROC curve for the multivariate logistic regression model was obtained with stepwise backward feature elimination, which included patients with the expander reconstruction method, postoperative chemotherapy (yes or no), type of mastectomy, and type of surgery (TM vs. RRM) was 0.83 (95% CI: 0.78-0.89), indicating a high predictive value for the occurrence of the composite endpoint.

These observations are in line with those given in previous studies [16,17]. Bettinger et al. showed higher expander and implant complications with obesity and advanced breast cancer often treated with chemotherapy and radiotherapy. Furthermore, univariate analysis showed that therapeutic mastectomy and contralateral prophylactic mastectomy at the same time led to a higher complication rate [16].

In our study, the type of mastectomy, implant or expander-based breast reconstruction, and the use of preoperative or postoperative chemotherapy and postoperative radiotherapy differed significantly between prophylactic and therapeutic surgery groups, which are limitations of our study. Mutation carriers in whom risk-reducing mastectomies (prophylactic surgery) were performed had significantly shorter hospitalization, better aesthetic outcomes, and lower complication rates as compared to breast cancer patients in whom therapeutic mastectomies were performed. More patients requiring RRM and NSM implant-based breast reconstruction had a prepectoral plane, which could indicate less aggressive treatment, shorter hospitalization time, and better patient-reported aesthetic outcomes.

We excluded patients with rippling (two cases) and capsular contracture (four cases) from our analysis owing to the fact that longer follow-up is needed [18,19].

In the present study, skin-reducing mastectomy in large, ptotic breasts and postoperative chemotherapy were associated with a composite endpoint comprising a higher complication rate and the presence of seroma. These predisposing factors were also reported by other authors [20]. Furthermore, postoperative chemotherapy increased the chance of both minor and major complications after breast reconstruction, while preoperative chemotherapy and postoperative radiotherapy increased the rate of minor complications. No statistically significant differences in terms of late major complications were found between the radiotherapy and no-radiotherapy groups in the present study; however, the follow-up time was short and the sample size of irradiated patients was small. Sigalove et al. reported that postmastectomy radiotherapy did not influence the significant increase in surgical complications for patients undergoing prepectoral breast reconstruction [8]. Indeed, long-term follow-up will be required to elucidate the true effectiveness of prosthetic prepectoral mesh-based breast reconstructions in the case of radiotherapy and the capsular contracture rate [16,21].

Previous studies indicate that the prepectoral technique is associated with less postoperative pain and the need for painkillers compared to the submuscular plane. However, our present findings do not suggest any statistically significant differences in postoperative pain between prepectoral and subpectoral cohorts. This could be explained by the fact that most of the subpectoral procedures (56.96%) used an expander and all (100%) used mesh, which reduced tension and thus pain. Nelson et al. report that prepectoral patients demonstrated lower pain on postoperative days 1 to 2 but no differences on days 3 to 10 [16]. Baker et al. have found that early postoperative pain and quality of life at 3 months are equivalent between groups. [17].

The finding of our study is that minor surgical complications (5.77% vs. 26.32%, *p* < 0.001) and the occurrence of seroma (20.51% vs. 40.79%, *p* = 0.001) were significantly less frequent in the prepectoral group than in the subpectoral group. Nelson et al. found an increased rate of seroma in the prepectoral group (prepectoral 16.9% vs. 3.4%; *p* < 0.001), likely because of a higher use of acellular dermal matrixes and an early learning curve relating to drain management. Then, their practice for prepectoral reconstruction drain removal has become more conservative, requiring output to be less than 30 cc for consecutive days before removal. Thus, the seroma rate decreased following this adjustment in postoperative care [22]. In our study, we used long-term resorbable synthetic meshes, not ADMs, and direct-to-implant breast reconstructions in 90.63% of prepectoral surgeries (Nelson et al. used expander-based breast reconstruction). We removed drains when less than 30 cc.

One of the major limitations of this study is its short follow-up. Other limitations of our study include non-homogeneity regarding BMI, age, radiotherapy, and chemotherapy. However, our findings are consistent with those of other investigators who compared the therapeutic and prophylactic, prepectoral, and subpectoral techniques in breast reconstructions [23,24,25,26]. Our results confirm that the mesh-based approach can be a safe technique. Nevertheless, prospective, multicenter, and long-term outcome data studies are needed to further evaluate the benefits of such treatments.

## 7. Conclusions

High body mass index (BMI), tobacco use, postoperative chemotherapy, expander-based breast reconstructions, skin-reducing mastectomies, and therapeutic mastectomies were linked to more adverse effects on breast reconstruction outcomes.

Patients in whom prepectoral surgeries were performed demonstrated significantly shorter median hospitalization time. They also were characterized by lower minor complication rates, but similar major late complication rates.

Implant-based breast reconstruction with the use of long-term, dual-stage, resorbable, synthetic mesh has emerged as an effective, safe method of breast restoration, associated with low morbidity and good cosmesis.

## Figures and Tables

**Table 1:** Demographic characteristics of individual patients participating in the study.

		Median (25–75%)			*p*
Variables		Prepectoral with Mesh (*n* = 109)	Subpectoral with Mesh (*n* = 61)	Min–Max	
BMI [kg/m^2^]	-	21.49 (19.87–23.02)	22.21 (21.10–24.77)	15.06–34.00	**0.015**
Age [Years]	-	42.00 (37.00–46.00)	46.00 (41.00–50.00)	24.00–74.00	**0.002**
Smoking	Yes	4 (3.67%)	5 (8.20%)	-	0.285
	No	105 (96.33%)	56 (91.80%)		

BMI—body mass index. Bold: Statistically significant.

**Table 2:** Patient and procedure characteristics (each case in the table denotes a single surgery) in relation to the type of surgery.

Variables		Prepectoral with Mesh (*n* = 156)	Subpectoral with Mesh (*n* = 76)	*p*
Breast	Left	83 (53.21%)	43 (56.58%)	0.628
	Right	73 (46.79%)	33 (43.42%)	
Weight of the specimen [g]	-	400.00 (330.00–475.50)	380.00 (320.00–445.00)	0.332
Size of the implant [mL]	-	375.00 (300.00–450.00)	385.00 (300.00–450.00)	0.950
Expander or implant placement	Implant	143 (91.67%)	31 (40.79%)	**<0.001**
	Expander	13 (8.33%)	45 (59.21%)	
Type of surgery	TM	41 (26.28%)	68 (89.47%)	**<0.001**
	RRM	115 (73.72%)	8 (10.53%)	
Type of mastectomy	SSM	5 (3.21%)	42 (55.26%)	**<0.001**
	NSM	151 (96.79%)	34 (44.74%)	
	SRM	16 (10.26%)	1 (1.32%)	**0.014**
Type of therapy	Preoperative chemotherapy	22 (14.10%)	46 (60.53%)	**<0.001**
	Postoperative chemotherapy	9 (5.77%)	10 (13.16%)	**0.095**
	Preoperative radiotherapy	6 (3.85%)	0 (0.00%)	0.181
	Postoperative radiotherapy	7 (4.49%)	17 (22.37%)	**<0.001**
Restriction of arm mobility after surgery	Yes	3 (1.92%)	4 (5.26%)	0.221
	No	153 (98.08%)	72 (94.74%)	
Patient-reported aesthetic outcome [scale 1–5]	-	5.00 (4.00–5.00)	4.00 (3.50–5.00)	**<0.001**
Hospitalization time [days]	-	4.00 (4.00–4.00)	4.00 (4.00–6.00)	**<0.001**
Postoperative pain [scale 0–10]	-	6.00 (4.00–8.00)	6.00 (4.00–7.00)	0.544
Minor complications	Yes	9 (5.77%)	20 (26.32%)	**<0.001**
	No	147 (94.23%)	56 (73.68%)	
Major Complications	Yes	17 (10.90%)	7 (9.21%)	0.869
	No	139 (89.10%)	69 (90.79%)	
Seroma	Yes	32 (20.51%)	31 (40.79%)	**0.001**
	No	124 (79.49%)	45 (59.21%)	
Stage *	0	4 (9.76%)	5 (7.35%)	0.154
	I	9 (21.95%)	15 (22.06%)	
	II	25 (60.98%)	48 (70.59%)	
	III	3 (7.32%)	0 (0.00%)	
Histological type *	IDC	36 (87.80%)	60 (88.24%)	0.807
	DCIS	4 (9.76%)	5 (7.35%)	
	ILC	1 (2.44%)	3 (4.41%)	

DCIS—Ductal carcinoma in situ; IDC—Invasive Ductal Carcinoma; ILC—Invasive Lobular Carcinoma; NSM—nipple-sparing mastectomy; SRM—skin-reducing mastectomy; RRM—risk-reducing mastectomy; SSM—skin-sparing mastectomy; TM—therapeutic mastectomy; * applies to the TM group only. Bold: Statistically significant.

**Table 3:** Surgery outcomes after therapeutic and risk-reducing mastectomies.

Variables	Median (25–75%)		*p*
	TM	RRM	
Hospitalization time [days]	4.00 (4.00–5.00)	4.00 (4.00–4.00)	**<0.001**
Postoperative pain [scale 0–10]	5.50 (4.00–7.00)	6.00 (4.00–8.00)	0.450
Patient-reported aesthetic outcome [scale 1–5]	4.00 (3.00–5.00)	5.00 (4.00–5.00)	**<0.001**

Bold: Statistically significant.

**Table 4:** Univariate logistic regression of factors potentially associated with the composite endpoint (postoperative minor and major complications including presence of seroma).

Variables	Effect Level	Coefficient	OR (95% CI)	*p*
Age [Years]	-	0.056	1.06 (1.02–1.09)	**<0.001**
BMI [kg/m^2^]	-	0.197	1.22 (1.11–1.33)	**<0.001**
Weight of the specimen [g]	-	0.002	1.00 (1.00–1.00)	0.131
Size of the implant [mL]	-	0.001	1.00 (0.99–1.00)	0.259
Smoking	Yes vs. No	2.869	17.63 (2.12–140.22)	**0.007**
Expander or implant placement	Expander vs. Implant	1.599	4.95 (2.62–9.37)	**<0.001**
Type of mastectomy	SSM vs. NSM	0.850	2.34 (1.22–4.48)	**0.010**
Type of mastectomy—SRM	Yes vs. No	1.762	5.83 (1.84–18.49)	**0.003**
Preoperative chemotherapy	Yes vs. No	1.092	2.98 (1.66–5.34)	**<0.001**
Postoperative chemotherapy	Yes vs. No	2.791	16.30 (3.67–72.50)	**<0.001**
Preoperative radiotherapy	Yes vs. No	1.180	3.26 (0.58–18.16)	0.178
Postoperative radiotherapy	Yes vs. No	0.889	2.43 (1.03–5.74)	**0.043**
Type of surgery	TM vs. RRM	1.583	4.87 (2.75–8.63)	**<0.001**
Histological type *	DCIS vs. IDC	0.959	2.61 (0.52–13.22)	0.247
	ILC vs. IDC	−1.392	0.25 (0.03–2.48)	0.235
Stage *	I vs. 0	−2.140	0.12 (0.02–0.71)	**0.020**
	II vs. 0	−0.661	0.52 (0.10–2.67)	0.431
	III vs. 0	0.560	0.57 (0.03–10.07)	0.702

BMI—body mass index; DCIS—Ductal carcinoma in situ; IDC—Invasive Ductal Carcinoma; ILC—Invasive Lobular Carcinoma; NSM—nipple-sparing mastectomy; RRM—risk-reducing mastectomy; SSM—skin-sparing mastectomy; TM—therapeutic mastectomy; * applies to the TM group only. Bold: Statistically significant.

**Table 5:** Multivariate logistic regression of factors potentially associated with achievement of the composite endpoint (minor and major complications after breast reconstruction including presence of seroma).

Variables	Effect Level	Coefficient	OR (95% CI)	*p*
Intercept	-	0.667	1.95 (0.05–77.45)	0.723
Age [Years]	-	0.031	1.03 (0.99–1.08)	0.152
BMI [kg/m^2^]	-	0.127	1.13 (1.00–1.30)	0.057
Weight of the specimen [g]	-	−0.003	1.00 (1.00–1.00)	0.267
Size of the implant [mL]	-	0.001	1.00 (1.00–1.01)	0.770
Smoking	Yes vs. No	1.177	10.53 (1.07–103.86)	**0.044**
Reconstruction methods	Expander vs. Implant	0.774	4.70 (1.39–15.91)	**0.013**
Type of mastectomy	SSM vs. NSM	−0.499	0.37 (0.11–1.21)	0.099
Type of mastectomy—SRM	Yes vs. No	1.277	12.86 (2.93–56.51)	**0.001**
Preoperative chemotherapy	Yes vs. No	0.154	1.36 (0.47–3.94)	0.571
Postoperative chemotherapy	Yes vs. No	1.408	16.72 (3.27–85.53)	**<0.001**
Preoperative radiotherapy	Yes vs. No	0.814	5.09 (0.56–46.58)	0.149
Postoperative radiotherapy	Yes vs. No	0.334	1.95 (0.62–6.10)	0.250
Type of surgery	TM vs. RRM	0.421	2.32 (0.79–6.82)	0.126

BMI—body mass index; NSM—nipple-sparing mastectomy; RRM—risk-reducing mastectomy; SSM—skin-sparing mastectomy; TM—therapeutic mastectomy. Bold: Statistically significant.

**Table 6:** Multivariate logistic stepwise backward regression of factors potentially associated with achievement of the composite endpoint (minor and major complications after breast reconstructions including presence of seroma).

Variables	Effect Level	Coefficient	OR (95% CI)	*p*
Intercept	-	1.180	3.26 (1.13–85.21)	0.479
BMI [kg/m^2^]	-	0.102	1.11 (0.99–1.24)	0.069
Reconstruction methods	Expander vs. Implant	0.744	4.43 (1.40–14.01)	**0.011**
Postoperative chemotherapy	Yes vs. No	1.278	12.89 (2.60–63.98)	**0.002**
Preoperative radiotherapy	Yes vs. No	0.845	5.42 (0.58–50.89)	0.139
Type of mastectomy—SRM	Yes vs. No	1.150	9.97 (2.52–39.35)	**0.001**
Type of mastectomy	SSM vs. NSM	−0.428	0.42 (0.13–1.34)	0.145
Smoking	Yes vs. No	1.055	8.25 (0.91–74.70)	0.060
Type of surgery	TM vs. RRM	0.704	4.08 (1.85–9.04)	**<0.001**

BMI—body mass index; RRM—risk-reducing mastectomy; TM—therapeutic mastectomy; SRM—skin-reducing mastectomy. Bold: Statistically significant.

## Data Availability

The data presented in this study are available on request from the corresponding author.

## Supplementary Materials

The following supporting information can be downloaded at: https://www.mdpi.com/article/10.3390/cancers14133188/s1. Table S1. Major postoperative complications among patients receiving pre- and/or postoperative therapy, Table S2. Minor postoperative complications among patients receiving pre- and/or postoperative therapy, Table S3. Multivariate logistic of all factors potentially associated with achievement of the composite endpoint (minor and major complications, including presence of seroma) for TM group, Table S4. Multivariate logistic of all factors potentially associated with achievement of the composite endpoint (minor and major complications, including presence of seroma) for RRM group.
