# Supplementary material for: A Retrospective Study Assessing the Outcomes of Immediate Prepectoral and Subpectoral Implant and Mesh-Based Breast Reconstruction
Source: Cancers (Basel). 2022 Jun 29;14(13):3188. doi: 10.3390/cancers14133188 (PMC9264839; doi:10.3390/cancers14133188)
Supplement: Supplementary file 1 [file cancers-14-03188-s001.zip › cancers-1729923-supplementary.pdf]

Table S1. Major postoperative complications among patients receiving pre- and / or postoperative therapy

Type of treatment	No major complication	Major complication	p
No preoperative chemiotherapy	151 (72.60%)	13 (54.17%)	0.101
Preoperative chemiotherapy	57 (27.40%)	11 (45.83%)	
No postoperative chemiotherapy	195 (93.75%)	18 (75.00%)	**0.005**
Postoperative chemiotherapy	13 (6.25%)	6 (25.00%)	
No preoperative radiotherapy	204 (98.08%)	22 (91.67%)	0.119
Preoperative radiotherapy	4 (1.92%)	2 (8.33%)	
No postoperative radiotherapy	188 (90.38%)	20 (83.33%)	0.287
Postoperative radiotherapy	20 (9.62%)	4 (16.67%)	

Bold: Statistically significant.

Table S2. Minor postoperative complications among patients receiving pre- and / or postoperative therapy

Type of treatment	No minor complication	Minor complication	p
No preoperative chemiotherapy	152 (74.88%)	12 (41.38%)	**<0.001**
Preoperative chemiotherapy	51 (25.12%)	17 (58.62%)	
No postoperative chemiotherapy	189 (93.10%)	24 (82.76%)	0.124
Postoperative chemiotherapy	14 (6.90%)	5 (17.24%)	
No preoperative radiotherapy	197 (97.04%)	29 (100.00%)	>0.999
Preoperative radiotherapy	6 (2.96%)	0 (0.00%)	
No postoperative radiotherapy	188 (92.61%)	20 (68.97%)	**<0.001**

Postoperative radiotherapy	15 (7.39%)	9 (31.03%)

Bold: Statistically significant.

Table S3. Multivariate logistic of all factors potentially associated with achievement of the composite endpoint (minor and major complications, including presence of seroma) for TM group

Variables	Effect level	Coefficient	OR (95% CI)	p
Intercept		-2.084	0.12 (0.00-14.61)	0.391
Age [Years]	-	-0.036	0.96 (0.91-1.02)	0.229
BMI [kg/m^2]	-	0.217	1.24 (1.03-1.49)	**0.022**
Weight of the specimen [g]	-	0.002	1.00 (0.99-1.01)	0.627
Size of the implant [ml]	-	-0.002	1.00 (0.99-1.01)	0.704
Smoking	Yes vs No	0.449	1.85 (0.53-6.43)	0.479
Expander or implant placement	Expander vs Implant	0.621	3.46 (0.86-13.92)	0.080
Type of mastectomy	SSM vs NSM	-0.559	0.33 (0.09-1.20)	0.092
Implant placement	Subpectoral vs Prepectoral	-0.126	0.78 (0.21-2.91)	0.707
Preoperative chemotherapy	Yes vs No	-0.417	0.4 (0.07-2.61)	0.362
Postoperative chemotherapy	Yes vs No	1.011	7.56 (1.23-46.42)	**0.029**
Postoeprative radiotherapy	Yes vs No	0.308	1.85 (0.53-6.43)	0.333
Histological type	DCIS vs IDC	1.857	6.41 (0.75-54.50)	0.050
	ILC vs IDC	-1.856	0.16 (0.00-5.26)	0.139
Stage	II/III vs 0/I	0.899	6.03 (0.95-38.33)	0.057

BMI – body mass index; DCIS – Ductal carcinoma in situ; IDC – Invasive Ductal Carcinoma; ILC – Invasive Lobular Carcinoma; NSM - nipple-sparing mastectomy; SSM – skin-sparing mastectomy.

Bold: Statistically significant.

Table S4. Multivariate logistic of all factors potentially associated with achievement of the composite endpoint (minor and major complications, including presence of seroma) for RRM group

Variables	Effect level	Coefficient	OR (95% CI)	p
Intercept	-	-9.584	0.00 (0.00-0.13)	**0.013**
Age [Years]	-	0.178	1.20 (0.09-1.32)	**<0.001**
BMI [kg/m^2]	-	0.200	1.22 (0.91-1.63)	0.175
Weight of the specimen [g]	-	-0.005	1.00 (1.00-1.00)	0.185
Size of the implant [ml]	-	-0.003	1.00 (0.99-1.00)	0.445
Expander or implant placement	Expander vs Implant	1.463	18.67 (1.28-272.98)	**0.032**
Type of mastectomy	SSM vs NSM	-2.006	0.02 (0.00-0.69)	**0.031**
Type of mastectomy - SRM	Yes vs No	1.817	37.86 (4.89-293.14)	**<0.001**
Implant placement	Subpectoral vs Prepectoral	0.507	2.76 (0.36-21.2)	0.330

Bold: Statistically significant.
